# Pathology and Treatment of Yellow Fever; with Some Remarks upon the Nature of Its Cause and Its Prevention

**Published:** 1881-06

**Authors:** H. D. Schmidt

**Affiliations:** New Orleans, La.


### ARTICLE II.

### THE PATHOLOGY AND TREATMENT OF YELLOW FEVER; WITH SOME REMARKS UPON THE NATURE OF ITS CAUSE AND ITS PREVENTION. By H. D. Schmidt, M. D., New Orleans, La.

(Continued from page 481, May No., 1881.)

### PATHOLOGICAL ANATOMY.

### 1. CONDITION OF THE SURFACE AND INTERNAL ORGANS OF THE BODY, AS REVEALED BY AUTOPSY.

As in the majority of fatal cases of yellow fever, the patient is attacked by the disease while in his ordinary condition of health, and, as in most instances only a few days intervene between the attack and the fatal issue, the dead body usually presents its natural appearance in form and dimensions. But, as regards the color, a most striking change has taken place; for the whole skin is now of a distinct orange-yellow color, even in cases where no jaundice was observed during the course of the disease. In fact, this yellow appearance of the skin is so invariably met with after death, as to have given rise to the very appropriate and characteristic name of the disease. The intensity of the color, of course, differs in different cases; but, it may be said, that it is usually proportionate to the severity of the case. Generally, it is deepest about the head and trunk, fading toward the feet, and corresponding to the manner of its progress during the course of the disease. Toward the end of an epidemic a number of cases are met with, in which the jaundice only extends to, or slightly beyond the knees. Very soon after death—sometimes even during the mortal agony—hypostatic congestions are observed, especially around the neck, shoulders and back ; but, frequently also about the face, ears, hands, feet, scrotum and penis.

In opening the *cranial cavity*, the *dura mater*, with the exception of the yellow tint which it presents, is usually found in a normal condition. In some instances, a smaller, or larger number of Pacchionian granulations, which, however, bear no relation to yellow fever, are met with. The serous fluid, contained in the arachnoid cavity, and also the surface of the brain covered by the arachnoid membrane and pia mater, present the same yellowish color. This coloration, moreover, is observed in all the tissues of the body, extent and intensity being proportionate to the severity of the case. The *pia mater* is almost always found more or less congested. Very frequently, not only the larger and smaller veins are filled with blood, but also the cerebral and basilar arteries with their arterioles. In some cases I have found the congestion to extend over the whole of the cerebrum and cerebellum, while in others it extended more or less only over the parietal and frontal lobes ; but, the pons varolii and medulla oblongata were invariably found in a state of considerable hyperæmia. The *arachnoid membrane* is frequently opaque, or even slightly thickened by exudation, and the subarachnoid space filled with serum. In such cases, the serous effusion has usually extended into the *substance* of the *brain*, producing an œdematous appearance of this organ. In most cases, however, the hyperæmia has extended throughout the whole of the latter, which then often appears swollen and œdematous throughout. The ventricles are then frequently found filled with a yellowish serous fluid, sometimes turbid in appearance, and the blood-vessels ramifying on their surfaces, together with the vessels of the choroid plexus, distended with blood. If the brain is cut into, the yellowish tint is observed to extend into its white or medullary substance, and the blood is seen issuing from the open ends of the cut blood-vessels. The blood-vessels of the pia mater of the cervical and lumbar portions of the *spinal*

*marrow*, also, are found congested with blood, particularly in the latter region ; in some cases, even, opacity of its arachnoid membrane is observed throughout the whole region.

In the thorax, the *lungs* generally present a normal appearance, though in some cases, smaller or larger portions of these organs are found congested, or in an emphysematous condition; other pathological changes met with do not properly belong to yellow fever. The *pericardium*, with the exception of the yellow tinge, is usually normal; but the fluid within its cavity, also tinged yellow, has sometimes appeared to be increased in quantity. In uncomplicated cases, the *heart* is generally found normal in size and form, and no changes are observed on its valves and tendinous structure, though the endocardium is frequently marked by the yellowish tint. In many cases, however, its muscular tissue, when cut, presents a pale yellowish hue, and is rather soft in consistence ; sometimes to such a degree as to be quite friable, and easily torn. In the latter instances, its reddish flesh color has entirely disappeared, and yielded to a pinkish yellow tint, indicating a high degree of fatty degeneration. In a number of cases, I met with yellow-tinged fibrinous bands, or clots of a delicate jelly-like consistence in the left ventricle ; or, also, with clots of coagulated blood in the cavities of the right side of the heart.

In opening the *abdomen*, the same yellow tint is presented by the organs enclosed in this cavity ; the liquid found in the latter is also frequently yellow. The veins of the abdominal viscera, contributing to the formation of the portal vein, are almost invariably found more or less distended with blood. In many cases, the congestion can be seen by the naked eye to extend into the venules of the intestines.

The *liver* invariably presents, either in parts or throughout, the characteristic pale yellow appearance, indicating fatty infiltration or degeneration. In former epidemics, I have met with a number of cases in which this yellowish appearance was only presented by larger or smaller portions of this organ, while others presented a bluish color, indicating a simple congestion; and again, smaller portions were observed to have retained their normal appearance. Moreover, when the organ was cut and pressed, small drops of bile were, in some instances, still observed issuing from the cut ends of the biliary ducts. During the epidemic of 1878, however, the fatty infiltration generally extended throughout the whole liver, often to such a degree as to render the parenchyma of the organ quite friable, and easy to be torn. In a limited number of cases, this organ presented more or less the well-known "nutmeg" appearance, while in others the congestion appeared to be confined to the "inter-lobular vessels."

The *gall bladder* contained, in almost every case, a greater or smaller quantity of a dark-brown, or black tar-like bile; its walls were thickened by œdema. Only in those cases in which the fatty infiltration had not extended throughout the whole liver, have I observed the bile of a lighter brownish color, and in a perfectly fluid condition.

The *mucous membrane* of the *stomach* presented in the thirty cases which I examined during the epidemic of 1878, more or less that peculiar form of congestion, to be described hereafter; and with only one exception, the stomach itself was found to contain a larger or smaller quantity of black vomit. In the exceptional case the patient had vomited the black fluid shortly before he died. As regards abrasions, or actual lesions of the mucous membrane of this organ, I have failed to discover any during the last epidemic, nor can I remember to have observed some in the cases which I examined in former epidemics. In only one case during the epidemic of 1878, I observed a black spot of an oval form, about 5 by 4 mm. in extent, in the mucous membrane, presenting a gangrenous appearance. On a closer examination, however, this proved to be an extravasation of blood into the tissue of the membrane, and not an actual lesion ; its surface being smooth and on the same level with the rest of the membrane. The color of this membrane, of course, depends on the extent and degree of intensity of the congestion ; though in those parts free from the latter it is generally normal in appearance. In those cases which I examined previously to 1878, I remember to have met with some in which the mucous membrane of the stomach presented but slight traces of congestion, and was unusually pale, but not friable, as has been stated by some authors. I cannot but think that the friable condition observed by the latter, was owing to post-mortem changes.

The *spleen* presents, in the great majority of cases, a normal appearance, and when cut its pulp assumes a scarlet hue. Only in exceptional cases did it appear enlarged, or did its pulp preserve its original dark, reddish-brown color.

The *kidneys* usually present a more or less abnormal appearance, both in consistence and color, though of normal size. In a few instances only I have observed them slightly enlarged, and softened. When a longitudinal section is made through one of these organs, it is found that the medullary substance, represented by straight tubules only, has preserved its flesh color, while the cortical substance has assumed a more or less yellowish tint. In a considerable number of cases, however, this tint does not extend throughout the whole organ, for there are certain portions left presenting an almost normal appearance. In fact, I have met, during the last epidemic, with cases in which the kidneys appeared entirely normal, so that in some instances I did not care to preserve them. In certain other cases during previous epidemics, I have observed the kidneys in a high degree of congestion, marked by a bluish-red color throughout. I have never met in yellow fever with the so-called "large white or smooth kidney," characteristic of parenchymatous nephritis.

The condition and appearance of the *supra-renal bodies* will be described in the following parts of this treatise.

The *urinary bladder* is generally found in a normal condition; in a few cases only I have observed traces of a slight hyperæmia in the mucous membrane, near the outlet of the organ. In about one-half of the cases examined in 1878, I found it still to contain a smaller or larger quantity of urine, and in one case it was even found distended with this fluid.

A peculiar slightly pungent odor, similar to that arising from the perspiration of the living patient during the course of the disease, may also be observed to arise from the interior of the dead body, while it is still warm, and when its cavities are opened, or when the muscles are laid bare and extensively cut into, as is done in removing the spinal marrow from its canal. This odor, like that arising from the body of the living patient, has at all times been perceived and mentioned by a number of physicians performing autopsies, while others have failed to detect it. The failure of the latter, however, may either have depended on a certain dullness of their olfactory sense, as once mentioned before, or upon their autopsies having been performed at a time when the body had entirely parted with its heat. In my own cases, the autopsies were made from three-quarters of an hour to three or four hours after death ; in only a few instances the time extended to five or six hours. I am the more convinced of the existence of this odor, as I have compared it with the ordinary odor arising from the dead bodies of cases dying from other diseases, and on which the autopsy was performed at the same time.

The above description of the condition of the organs of the dead body in fatal cases of yellow fever, as revealed by macroscopical examination, relates in particular to the thirty autopsies which I carefully made during the epidemic of 1878; and, furthermore, to others almost as numerous, and performed at the Charity Hospital during previous epidemics, either by myself or by the surgeon or resident students of that institution.

### 2. Pathological Changes, Occurring in the Tissues of the Organs during the Course of the Disease, and Revealed by Microscopical Examination.

The pathological changes produced in the tissues of a number of organs by the direct or indirect action of the noxious poison of yellow fever, are, individually considered, by no means pathognomonic of this disease. On the contrary, they are equally observed in other diseases, whether infectious or not, in which the nutrition of the system has been disturbed and deranged, either by the interference of an infectious poison, or by the abnormal performance of the function of one or another organ. It is, therefore, only when considered as a complex, and in association with the clinical, phenomena, that these changes may receive a pathognomonic significance. But, even, without regard to this significance, the exact knowledge of these changes is so highly essential to the knowledge of the true nature of the disease, as to render the importance of their microscopical study obvious to every physician. For this reason, I shall, in describing the pathological condition of the organs, as revealed by a close and accurate microscopical examination, enter more into the details of the subject than I have done in the preceding part of this treatise, in which the object in view was to combine exactitude of description with brevity of communication. And as this treatise is intended to be read, not only by the accomplished physician, but also by the medical student, as yet not thoroughly familiar with the more minute studies of anatomical science, I deem it practical to draw a brief sketch of the normal histology of the respective organs, before describing the pathological changes which they undergo during the course of the disease.

I shall begin with that most important and interesting tissue, the blood, which, partly solid, partly fluid, receives all matters, essential to the maintenance of the life of the body, and which, in distributing them to all its parts, represents the most important factor in the process of nutrition. But, while it thus receives and distributes the materials essential to the normal condition of the organism, it is also capable of receiving noxious substances, such as infectious poisons, from the surrounding air, which, in disturbing the process of nutrition by their deleterious influence, give rise to pathological phenomena, characteristic of the disease under discussion. The condition and behavior of the morphological elements of this liquid tissue in a number of diseases have of late formed a special subject of research and discussion, and have given rise to many contradictory statements and views. It is therefore desirable for the medical practitioner to be acquainted with the leading facts of this subject, to enable him to form an opinion of his own.

### THE BLOOD.*

The colored blood corpuscles of man represent, like those of other mammalia, minute bi-concave disks, the mean diameter of which ranges, according to the very accurate measurements of *Woodward*, from .00731 to .00772 mm., though in some speci-

* A portion of the following description of the morphological elements of the blood will be taken from my last paper on this subject, embodying my own researches, viz.: "The Structure of the Colored Blood-corpuscles of Amphiuma tridactylum, the Frog and Man," published in the *Journal of the Royal Microscopical Society of London*, 1878.

mens I have observed them slightly larger. They are very delicate in substance, and being elastic and flexible in an unusually high degree, are enabled to resume always their original form when distorted by mechanical causes. In fact, the momentary changes of form, which they are constantly undergoing when floating in the liquor sanguinis, are owing to the great delicacy and elasticity of their protoplasm. In examining them under the microscope with a sufficient amplification, we observe that the most feeble current arising in the liquid in which they float, disturbs their form, either directly, or from mutual contact or pressure. When a colored blood corpuscle of man is examined in a state of rest from the front, its outline appears perfectly round. Bring it into proper focus, so that its outlines appear the most distinct, its center, to the extent of about one-third of the whole diameter appears light. Proceeding toward the periphery of the corpuscle, a slight shade is seen to arise from the light center, which, after increasing somewhat in depth, is gradually lost, to be followed by the high light, representing the convexity of the margin. This variation of light and shade is, of course, caused by the form of the corpuscle. The convex margin appears most illuminated at the highest part of the convexity, when the rays of light passing through it undergo very little refraction, while more or less shade must appear, by virtue of the refraction of light, at that part of the surface which inclines toward the center, and forming a part of the concavity; the center, finally, being the thinnest portion of the corpuscle, and very nearly flat, must, from the absence of almost any refraction, appear light. Owing to the rounded margin of the blood corpuscle, its very outlines do not appear distinct and sharply defined; on the contrary, a delicate shade is observed at the very edge, which soon disappears in the high light of the convexity. No trace of the existence of a membrane, or a membranous layer can be discovered in the fresh blood corpuscle of man. When brought into exact focus, it appears encircled by a narrow ring of a pinkish tint, much lighter than the rest of the surrounding liquor sanguinis; this phenomenon is probably owing to the corpuscle refracting the light towards its less refractive medium. The appearance of the colored blood corpuscle of man in exact profile is peculiar, and corresponds not to the bi-concave form in which it is so often erroneously represented. Recollecting that its body represents a minute plate or disc, the peripheral portion of which, besides being convex and rounded at its border, is twice as thick as the central, which is concave; and further, that it is perfectly symmetrical at all points, it becomes evident that the outlines of its profile must be represented by two *straight* and *parallel* lines, connected at their extremities by two semicircular ones; and accordingly the side-view of the corpuscle could not reveal the concavity of the central portion. But, if a vertical section were made through the center of the corpuscle, the outlines of the cut surface would be represented by two slightly curved lines, which, directing their convexity toward each other, are connected by semicircular lines, the whole showing not only the bi-concave form of the central, but also the convex and rounded form of the peripheral portion of the body.

The color of a single blood corpuscle when seen under the microscope is not yellow, as has been stated sometimes, but more of a light greenish tint. It is only when a number of blood corpuscles are collected into a small mass or group, and rest upon each other, that the original greenish tint merges into a yellow. With the increase of the thickness of the mass, the yellow becomes darker, and finally passes into the scarlet red, as seen in a drop of blood.

Soon after a small drop of human blood has been put on the glass slide and covered with the plate of thin glass, a number of blood corpuscles are observed to change in various manners, and in a slighter or greater degree, their original form. In some instances the convex peripheral portion of the corpuscle appears to shrink in thickness at one point, while the rest of it swells and gains in diameter, so that the whole body assumes a wedge-like form. In other instances the blood corpuscle assumes the form of a shallow basin, an appearance which is very probably caused by a contraction of the convex border of only *one* surface, by which process the concavity of this surface increases in depth, while the other surface is rendered convex instead of concave. If the contraction continues, the corpuscle assumes the form of a deep cup. Whether during this process the central portion increases in thickness, while the convex periphery is rendered thinner by the contraction, is difficult to determine; but that the contraction takes place only on *one* surface of the convex margin is obvious, for, if it occurred throughout the whole margin, the corpuscle would not become cup-shaped, but would preserve its original form of a disc, though its central portion might gain in thickness, while the concavities of its surfaces would decrease in depth, or entirely disappear. Sometimes the convexity of these cup-shaped forms terminates in a conical protuberance. There is another very singular form observed, difficult to describe, but resembling in some respects the wedge-like form, with the exception of the narrow portion of the wedge; instead of being straight, assuming here the form of a Y, showing that the body must be three-sided.

Intermediate forms, similar to those above described, are observed; but it will be noticed that in all these instances it is only a portion of the protoplasm of the blood-corpuscle which is contracting, while the rest is expanding in compensation of the contraction. There are, however, other changes occurring in the form of the corpuscles, owing to a contraction of the protoplasm throughout the whole body, and accompanied by a considerable diminution in size. As the result of such a contraction we may regard those familiar forms of blood corpuscles, generally compared to a mulberry or thornapple. These, when occurring on the blood corpuscle of fresh blood, and without the action of a reagent, have generally been attributed to the evaporation of the liquor sanguinis. With the increase of the density of this liquid, namely, an exosmotic current from the blood corpuscle is supposed to be induced, causing shriveling of these bodies. If this view were correct, those corpuscles nearest to the edge of the film or layer of blood under the covering-glass, should first undergo the changes in question. But this is not always the case; on the contrary, single mulberry or thornapple-shaped blood corpuscles are observed among the mass of unaltered ones in the center of the preparation, immediately or soon after the blood is placed on the slide. Soon after, other individual corpuscles are seen to assume these forms, the number increasing with the time, until finally whole groups or the entire mass may undergo this change. In other instances, considerable portions of the blood corpuscles of the preparation may, soon after the blood is put upon the slide, contract at once, and assume the thornapple form, appearing almost as if affected by a general contagion. The thornapple form may even be produced by the action of water, as I have observed. The form of the blood corpuscle, when gradually and slowly undergoing these changes, seems to pass through several phases. The first deviation from the original form observed consists in minute elevations and protuberances, arising from the surface of the corpuscle at its rounded margin, thence, while increasing in number, extending over the entire corpuscle. Next, the protuberances, at first of a conical form, become gradually smaller in diameter at their base, while their points or summits enlarge to assume the form of an imperfect knob, resembling a granule. It is at this stage of the change that the form of the blood corpuscle has been compared to that of a mulberry. As the change continues the globular projections become thinner, until finally they are transformed into minute, sharp, spinous processes. In this state the blood corpuscle resembles a thornapple. But it is not necessary that, when the change of form has set in, it should pass through all the stages to the ultimate thornapple form; on the contrary, the contraction may cease at any stage of the process, or even cause at once the mulberry or thornapple form. Very little alteration in the general form of the corpuscle is observed to take place by this process; it is not rendered spherical, as might be supposed, but represents still a disc, though the concavities may have disappeared from its surfaces, or even be converted into convexities.

The colored blood corpuscles of man are also endowed with the power of spontaneous motion, protruding and *retracting* minute processes similar to those observed on the thornapple form, a phenomenon which I first discovered in the summer of 1871, and which shows that these bodies not only possess a certain inherent power of contracting their bodies, but also of resuming their original form by a subsequent expansion, a characteristic property of the living protoplasm, enabling the corpuscle to manifest these motions, though not to so great an extent as is seen in the colorless. Keeping in mind this inherent property of the colored blood corpuscles, a part of the difficulty hitherto encountered in tracing these various spontaneous changes, occurring in the form of these bodies, to their cause, will be removed.

Nevertheless, as these changes are not only observed to occur in different localities of the same preparation of blood, and under different circumstances, but, moreover, are observed to occur in a greater or less degree in different specimens of blood taken at different times from the same individual, much doubt will still remain as to the true cause of the phenomenon, and the conditions under which it is manifested. The question, therefore, arises: Do these changes of form indicate progression and development, and a high degree of vitality residing in those blood corpuscles exhibiting them, or do they result from a loss of vitality, and are they manifestations of retrogression and decay? In the one case, they would probably occur on the young, in the other on the old corpuscle.

If the changes affecting the form of the colored blood corpuscle, and evidently caused by a contraction of its protoplasm, were indicative of a higher degree of vitality or molecular action, we should meet with a greater number of mulberry or thorn-apple-shaped, or otherwise deformed corpuscles in the fresh blood. But as, on the contrary, the number of these forms is comparatively small, immediately as the blood is removed from the living tissues, and, moreover, increases sometimes quite rapidly in proportion to the length of time intervening, it appears more probable that these changes of form indicate retrogression, and we may be justified in regarding them as the result of the last vital action manifested by the blood corpuscle, and portending its death.

When a very small portion of human blood is spread upon a glass slide, and after being covered by a thin plate of glass, a drop of water is added to the preparation, it will dilute the liquor sanguinis and induce an immediate escape of the hæmoglobin from the blood corpuscles, rendering the liquid in the vicinity of the latter, according to their number, more or less turbid. At the same time the blood corpuscles will be set in motion, and float away in the clearer parts of the liquid. Continuing to part with their coloring matter, they are gradually rendered pale, and finally appear as mere shadows. If now a portion of the liquid is removed by the careful application of a minute point of a piece of blotting paper, and its place filled by a small drop of clear water, the shadow-like blood corpuscle—if examined by a first-class objective—will appear bordered by a delicate double contour. The central portion of the corpuscle, encircled by the inner contour, appears now of the color of the field, while the margin, included by the two contours, appears lighter when put into the exact focus. This double contoured margin represents the outer portion of the protoplasm of the colored blood corpuscle forming the surface, which is denser in its molecular constitution than the rest of this substance, enabling it to withstand the solvent action of the water, endosmosing into the interior of the blood corpuscle, while the rest of the protoplasm is discolored, and perhaps dissolved, or otherwise affected by the action of this fluid. In the fresh and unaltered condition of the blood corpuscle of man, this denser portion of the protoplasm, forming a very thin layer on the surface of the latter, cannot be demonstrated, having the same index of refraction as the rest, but by the action of water, or of solutions of certain other reagents, it becomes visible in the form of an artificial or pseudo-membrane, for which reason I have named it the "membranous layer" of the blood corpuscle.

The changes produced in the colored blood corpuscle by the action of water are equally observed when these bodies are treated by a number of other reagents, both in liquid and gaseous form; they mainly consist in the escape of the hæmoglobin, contraction or coagulation of the protoplasm, and change of form. The action of the chloroform vapor particularly is very rapid in causing the escape of the hæmoglobin, though it does not dissolve the corpuscles as has been stated.

As regards the colorless blood corpuscles of man, it may be stated that they consist of a pale, finely granular protoplasm, inclosing, in most instances, one homogeneous disc-shaped nucleus, though sometimes two nuclei are observed. In the fresh blood, a considerable difference exists in the size of these corpuscles. In the protoplasm of the larger corpuscles, a group of dark-bordered granules, much larger than those properly belonging to the protoplasm itself, and rather similar to the pigment granules of the gangloin cells of the nervous centers, are observed. If a specimen of blood is examined fresh, and under the proper condition, these dark granules may be observed to perform certain oscillating movements, while the pale granules of the protoplasm also exhibit a constant motion, though more rotary or molecular in kind, and not as active as that of the former. Besides these movements of the individual granules—which seem to be identical with the well-known "Brownian movements" of minute particles—there are the so-called amœboid movements, in which the entire protoplasm of the colorless blood corpuscle takes part, consisting in a continuous change of form by the alternate projection and withdrawal of larger or smaller processes, resulting in locomotion. The amœboid movements of the protoplasm are not regularly observed to take place on all colorless blood corpuscles of the same specimen of blood; according to my own observations they appear to occur more rarely in the smaller individuals than in the larger. In some cases, these movements appear immediately after the fresh drop of blood is put upon the glass slide; in others, again, only after the addition of some water or slightly alkaline solution, or the application of warmth. In the fresh unaltered condition no enveloping membrane can be discovered on these corpuscles, but by the action of water I have frequently observed them bounded by a very delicate double contour. The proportionate number of the colorless blood corpuscles to the colored is very small, and varies in the blood vessels of different parts of the body of the same individual, as well as at different periods of the day. In the normal condition of the blood, the proportion has been estimated to be one colorless to two or three hundred colored blood corpuscles.

Besides the above described blood corpuscles, an insignificant number of very minute colored corpuscles are met with in many specimens of normal human blood. The diameter of these bodies ranges from hardly one-third to two-thirds of that of the ordinary colored blood corpuscles. They have been called "microcytes," and regarded as younger individuals; a view corroborated by my own observations on the development of the colored blood corpuscles in the human embryo, where many of them are met with in the blood as late as the third and fourth month of embryonic life.* The same view is taken by *Hayem*,† who met with the smallest forms of these bodies, $\frac{2}{1000}$ mm. in diameter, always under conditions where a new formation of colored blood corpuscles might be supposed, as in new-born children, menstruating women, after losses of blood, or in feeble individuals. I have frequently observed these minute corpuscles having assumed the thornapple form.

Independent of these minute colored corpuscles, however, not unfrequently other elements are met with in normal blood, not larger than minute organic granules, and colorless. They have been seen and described under different names by different observers, and have been generally considered to be derived from the granular protoplasm of the colorless blood corpuscle. When first known as the "elementary bodies" of *Zimmermann*,‡ who had demonstrated them in salted blood, they were regarded as the colorless remains of disintegrated colored blood corpuscles (Henson), and to be identical with the granular formations, previously met with in the blood by *Max Schultze*; *Béchamp* and *Estor*, who also observed these granular elements in the blood, called them "microzyma;" and, observing under the microscope a colored blood corpuscle breaking up into a number of these microzyma, while, on the other hand, they also observed a number of these elements aggregating and forming a colorless blood corpuscle, they concluded that all blood corpuscles represented aggregates of microscopical organisms.§ Elements, identical with the above, were moreover mentioned by *Bettelheim* and *Lostorffer*. Under the name of "hæmococci," they were subsequently described by *Nedsvetzki*‖ as minute round bodies, resembling the granular particles of the colorless blood corpuscles, and forming normal constituents of human blood, becoming very distinct with an amplification of from 900 to 1,000 diameters, and found in large numbers among the colored and colorless corpuscles. In the dead colorless blood corpuscles, he observed molecular motion to take place, and, furthermore, the formation of a light zone

**Monthly Microscopical Journal*, February, 1874.

†*Virchow u. Hirsch*, Jahresbericht fuer das Jahr, 1877, Vol. I., p. 38.

‡ *Rollett*,—Stricker's Handbuch der Lehre von den Geweben, etc., p. 300.

§ *Virchow u. Hirsch*, Jahresbericht f. d. Jahr 1870, Vol. I, p. 18.

‖ l. c.—für das Jahr 1873, Vol. I, p. 50.

around them, into which the minute granules entered, and thence passed into the liquor sanguinis, where they continued their movements in the same manner as in the hæmococci.

At present, there remains no doubt but that all these granular elements, met with in normal blood, and named differently by different observers, are derived from the finely granular protoplasm of the colorless blood corpuscles, and the movements they may exhibit are simply "Brownian" in their nature. I have frequently met with protoplasmatic remains of colorless blood corpuscles in human blood; and, a number of years ago, in the case of a man poisoned by cyanide of potassium, I observed, eight hours after death, a number of these amœba-form masses of protoplasm without nuclei, in which the minute pale granules were still in active motion; which continued for a considerable time after the addition of water to the preparation.

Having, by the above sketch of the anatomical elements of the human blood, prepared the way for the description and discussion of the condition and behavior of these elements in yellow fever, I shall now return to the original subject. The specimens of blood which I examined during the epidemic of 1867, were obtained from hæmorrhages from the nose, occurring on some of my patients. The blood was collected in small vials, and a limited space of time, of course, elapsed, before I could examine it at my office. The microscopical examination of these specimens showed that the greater portion of the colored blood corpuscles had assumed the thornapple form, but I was unable to detect any other abnormal condition of these bodies, nor any foreign body in the specimens. In other specimens—taken during the same or succeeding years—after death from the larger superficial veins of patients at the Charity Hospital, I was also unable to detect anything foreign to this liquid. The blood corpuscles, in general, exhibited the same characters and phenomena as those of healthy blood under the same conditions. Only in one case, I observed some phenomena on a small number of colored corpuscles, which, at that time, I was unable to explain satisfactorily to my mind. These blood corpuscles, namely, presented two or three *apparent* openings, or, better said, solutions of continuity upon their surfaces, manifesting themselves by a bright light-pinkish color.

From what I know at present, this phenomenon must have been owing to the presence of minute vacuoles in the protoplasm of the corpuscles, such as I, sometime afterward, discovered on a large scale in the giant blood corpuscles of *Amphiuma tridactylum.* On some other colored corpuscles of the same specimen, I observed the membranous layer of the corpuscle separated from the rest of the protoplasm, a phenomenon which I also subsequently observed on the colored blood corpuscles of the frog. As, in these cases, the blood had been taken from the dead body, it is obvious that the phenomena observed were owing to the post-mortem changes, for which reason I attached no importance to the observation.

During the month of August, in the midst of the epidemic of 1878, I made, more systematically than before, a series of examinations of the blood of living patients at the Charity Hospital. They were made in the following manner: The microscope being placed near one of the windows of the ward for the purpose of obtaining the best light, and with everything required for the examination, such as glass slips, covering glasses, etc., in perfect order and readiness, I went to the bed of the patient, and to obtain the blood made a small incision into his forearm. Knowing from my former researches upon the normal blood that the colored blood corpuscles of the first drop, issuing from the capillaries of the skin, are more liable to changes of form, when placed upon the glass slide, than those coming from deeper parts, I took the precaution of making no use of the first drop, except in a few instances. For this reason, the skin was again cleanly wiped, and a small portion of the blood next appearing in the incision quickly—at the bedside—placed upon the glass slip, and covered with a very thin and small cover glass. As soon as covered, it was put upon the microscope for examination.

Previous to these examinations of the blood of the living patient, I had observed in some sections of liver and of some other organs, taken from yellow fever cases, extravasations of free hæmoglobin from the capillary vessels, and its absorption by the surrounding cells; and, in accordance with this observation, I somewhat expected to meet with free hæmoglobin in the blood itself, supposing its escape from the colored blood corpuscles to be caused by the action of the specific poison of the disease upon the latter. And, by coincidence, I really found in the first specimen which I examined some of this coloring material, which, however—as must be remembered—is a phenomenon not unfrequently met with, even, in the examination of normal human blood. In this specimen, moreover, I observed that the greater portion of the colored blood corpuscles presented mulberry and thornapple forms, while the rest was rapidly assuming them. In all other respects, with the exception of a few microcytes, the blood appeared perfectly normal. *Not a single bacterium, or spore of a fungus*, was I able to discover, though I honestly endeavored to do so. In the next specimen of blood which I examined, and which was obtained from the same patient and from the same incision, I did not even meet with the free hæmoglobin. But, as in the first specimen, in this also, the greater portion of the colored blood corpuscles had already assumed the mulberry or thornapple forms when first seen under the microscope, while the rest was undergoing these changes quite rapidly.

In this manner I examined the blood of *fifteen* living patients, representing the different stages of the disease : that is, from the second day to one-half hour before death, or to convalescence. Two specimens of blood were examined in each case; in some even as many as three or four, but on different days. In all the cases, with the exception of two, the blood corpuscles presented the mulberry and thornapple forms, adhering frequently to each other in the form of rolls, forming irregular anastomoses. In a number of these cases the blood corpuscles had already assumed these forms and arrangement when first seen under the microscope; this was decidedly the case in a specimen of blood taken from a patient during the agony of death. In the two cases of exception, the colored blood corpuscles presented an appearance as normal in form and character as I ever beheld, and, moreover, retained it for a considerable time. The patients here were both Germans; the one, a boy, of 15 years, who had arrived at New Orleans by an English steamer three weeks before he was taken with the disease; his blood was examined on the fourth day, during the second stage of the disease; he recovered. The other was a strong and hardy young man of 22 years, a gardener, who had arrived from Germany during the month of February, 1878; his blood was examined on the second day of the disease, when he was very restless and slightly delirious; he died on the fifth day.

As regards the colorless blood corpuscles in the specimens of blood taken from the above-mentioned fifteen patients, nothing specially abnormal could be discovered. In one specimen only, taken from the blood oozing from a slight wound of the ear of a patient, and during the second stage of the disease, the relative number of these corpuscles appeared to have slightly increased, and amœboid movements were observed on some of them. In the other specimens, most of the colorless corpuscles observed belonged to the smaller kind, performing no amœboid movements, their number being, as far as I could judge, in a normal proportion to the colored blood corpuscles.*

Two specimens of blood, obtained two hours after death, for the purpose of determining the relative quantity of fat they contained—the one from the portal vein, the other from the right side of the heart—were also examined. They presented nothing remarkable. A portion of the colored blood corpuscles had assumed the thornapple form, while the rest had preserved their natural appearance; they were adhering to each other, forming rolls, no bacteria or fungi-spores were met with.

In reviewing now the results of my numerous examinations of the blood, it must be admitted that the condition in which its morphological elements were found, actually offers nothing remarkable, or otherwise, which in any way could be interpreted as peculiar or characteristic of yellow fever. The only conclusion to be drawn from these observations is, that the colored blood corpuscles of yellow fever blood are strongly inclined to assume the mulberry and thornapple forms as soon as removed from the circulation, indicating, in accordance with the view I have previously expressed, a certain loss of vitality. But the significance,

*The results of my examinations of the blood in yellow fever have been corroborated by *Dr. Sternberg*, who examined a considerable number of specimens in the Military Hospital at Havanna in 1879, without discovering any bacteria, or other minute organisms, in the fresh blood. Regarding the colorless blood corpuscles, however, he met in several cases with some which presented fat globules in their protoplasm, a phenomenon which finds a satisfactory explanation in the fatty infiltration of other organs.

which this phenomenon might appear to have in connection with the particular nature of yellow fever, is lost when we consider that the same behavior of the colored blood corpuscles has been noticed in the blood of patients laboring under other febrile infectious diseases by a few observers, though others fail to mention it. Thus, *Laptschinsky** found in his histological examinations or the blood of patients, suffering especially from febrile infectious diseases, that the colored blood corpuscles formed no columns of so-called money-rolls, but aggregated in masses and lumps of different sizes and form. The individual blood corpuscles frequently appeared as if swollen and turbid, their contours being less distinct. In such cases, he also frequently met with very minute blood corpuscles, adhering to each other quite tenaciously in the form of small aggregates. The colorless corpuscles, also, appeared increased in number, their amœboid movements were distinct, and extended upon the nuclei.

Thus far, then, no particular value should be attached to the fact that, with a few exceptions, the colored corpuscles of yellow fever blood assume the mulberry or thornapple form quite rapidly after their removal from the circulation; the significance of this phenomenon should be regarded as of secondary importance. Although I presume that the cause of this unusual tendency of the colored blood corpuscles to assume these abnormal forms is probably due to a loss of vitality by the pernicious influence of the infectious poison, I do not attribute the final effect to this cause alone, but, on the contrary, rather consider their removal from the living tissue, together with the exposure to the atmosphere, as the main factors concerned in the whole phenomenon. The loss of vitality renders the blood corpuscle more sensitive to the influence of these factors, which, in its healthy condition, it might have resisted for a limited space of time. In a number of specimens of blood, taken from the living patient, a portion of the colored blood corpuscles, as above mentioned, had already assumed these abnormal forms when first seen under the microscope; an observation which might lead to the supposition that they had undergone this change while still circulating in the blood vessels. I can safely assert that this is not the case, for I

**Virchow u. Hirsch*, Jahresberischt f. d. Jahr. 1874, Vol. I., p. 339.

do not remember that in the numerous thin sections of all parts of the brain and other organs, which I have made and examined, and in many of which—especially in those of the brain—all the minute blood vessels are filled with blood, I have ever met with mulberry or thornapple-shaped blood corpuscles. And, moreover, in order to satisfy my mind on this particular point of the subject, I have lately re-examined a very considerable number of thin sections of the brain, sympathetic ganglia, and some other organs, and obtained the same results.

But though we may not attach any pathognomonic value to these almost constantly occurring morphological changes in the colored blood corpuscles in yellow fever, they may nevertheless be regarded as a result of the febrile process in general. Of late years, quite a number of observers have directed their attention, not only to the relative proportion of the morphological elements of the blood to the liquor sanguinis, or to the relative number of the colored and colorless blood corpuscles themselves, in various diseases of the blood, but, moreover, to the morphological changes to which these bodies are liable in different diseases. The extreme sensitiveness of the blood corpuscles to extraneous influences, however, renders it in many cases difficult to determine whether the changes observed are due to the latter, or to real morbid causes; therefore, the investigator should be perfectly familiar with the morphological changes observed in normal blood, and not be endowed with a too vivid imagination.

As regards those minute colored corpusles, the so-called "microcytes," the proportionate number of which in normal human blood is 1 to 2000 of the ordinary colored blood corpuscles, they may also increase in number in certain diseases of the spleen and liver. *Vanlair* and *Masins** met with an extraordinary case of this kind, in the blood of which (a woman) they found the number of the microcytes increased to such an enormous extent, as to have only two colored blood corpuscles to 100 microcytes. This case induced the authors to apply the particular name of "microcythæmia" to this affection.

Those minute granular colorless elements, the "microzyma" of Béchamp, resulting from the disintegration of colorless blood

*L. c.—fuer das Jahr, 1872, Vol. I., p. 201.

corpuscles, may also, under certain pathological conditions, be found increased in quantity in the blood. They have not unfrequently been found, as well in febrile as in non-febrile diseases, and may give rise to very serious errors, by being mistaken for bacteria. I am much inclined to think that the statements of some physicians, relating to the presence of bacteria in yellow fever blood, were based upon such observations; or, if the bodies observed were bacteria in reality, they certainly entered the blood sometime after it had been removed from the living or dead body.

THE HEART AND LUNGS.

The observations upon these organs worthy of any remark, have already been made in the section treating of their condition as revealed by the autopsy. As regards the heart, it may be added that in those cases in which a fatty degeneration of this organ exists, the degenerative process is very rarely observed to have extended throughout the entire organ, but is mostly found limited to certain portions. The process itself does not consist in a mere infiltration of fat, as is met with in the liver, but in a true fatty metamorphosis of the muscular fibers. As fatty degeneration of the muscular tissue of the heart, however, not unfrequently takes place during the course of other infectious diseases, such as typhus, etc., and even in some other diseases of a chronic character, it cannot be regarded as pathognomonic of yellow fever.*

The *lungs* are, as has been already stated, generally found in a normal condition. In a few exceptional cases, small portions of them are found in a congested, or, also, emphysematous condition. But in consideration of these changes being observed but very rarely, they should be regarded as of an accidental origin, bearing no special relation to yellow fever. It is rather the immunity from the noxious influences of the yellow fever poison which these

* In the report of the "Havanna Yellow Fever Commission of the National Board of Health," it is stated: "that there is no foundation for the opinion that there is a fatty degeneration of the muscular fiber of the heart in yellow fever." This assertion can only be explained upon the supposition that the hearts examined very singularly *happened* to belong to such cases in which the fatty degeneration of this organ was really absent, as it is hardly probable that the type of the "yellow fever at Havanna" should so much differ from that of New Orleans; or that the expert who made the examinations, could have failed to recognize this well-known pathological fact, observed in many cases of this disease.

organs manifest, that may be considered as a characteristic feature of the disease.

THE LIVER.

The pathological changes occurring on the parenchyma of this organ, are so invariably met with in fatal cases of yellow fever, that they may safely be looked upon as a pathognomonic feature of the disease; and they should, therefore, be studied with the greatest care and attention. The close relationship in which the liver stands through its portal circulation with other abdominal organs, renders the latter liable to be involved in almost all the disorders of the former, a complication very frequently occurring in yellow fever. In such cases an approximate idea of the condition of the liver is easily obtained by observing those clinical symptoms indicating the condition of the stomach and intestines, and which may prove of service in the prognosis of the case; for it may well be presumed that the general condition of the patient very nearly corresponds to the extent of the pathological changes taking place in the liver. Thus it is, that in most fatal cases of this disease, the pathological changes revealed by the macroscopical and microscopical post-mortem examinations in the liver, are almost always found to exist in a high degree, a circumstance which leaves little opportunity to the pathologist of studying the initiary stage of the pathological process.

From the hyperæmic condition in which a number of the other organs of the body are found after death, it may also be presumed that the pathological changes observed in the liver are likewise preceded by a state of congestion. Although in all the cases which I examined during the epidemic of 1878, the liver was found affected with fatty infiltration throughout its whole extent, as already mentioned, the above view is sustained by those cases which I observed in previous epidemics, and in which portions of this organ were still found in a state of hyperæmia, preceding that of fatty infiltration or degeneration.

But, while the process of fatty infiltration of the liver is found to have taken place in all fatal cases, and thus forms a typical character of the disease, certain traces of other pathological processes are also discovered to have occurred in this organ in a number of instances, indicating probably a severer form, or other complications of the disease. In the following description of the changes in the parenchyma of the liver, I shall, therefore, begin with that particular condition, observed in the majority of cases, and representing the general type.

When, in these cases, a thin section of the fresh organ is made and examined in water under the microscope, the hepatic cells appear almost completely hidden by larger or smaller fat-globules, and to such an extent as to render the application of ether to the preparation necessary, in order to get a fair view of its component anatomical elements. After the section is cleared up by the ether dissolving the free fat, the hepatic cells will be found more or less distended by a considerable number of fat-globules. The size and quantity of these globules differ in different cells, for while some of the latter may be distended by one very large globule occupying almost the whole interior of the cell, and surrounded by a thin layer of its protoplasm, others may, besides a few larger globules, also contain a more considerable number of smaller ones. The nucleus is in most cells hidden by the fat. If one of the terminal branches of the hepatic veins—intra-lobular—should be contained in the section or fragment under examination, it will frequently be found that the cells in its immediate vicinity present more or less a greenish-brown color, apparently owing to the presence of bile-pigment. Thus far, the examination of the fresh specimen reveals but little beyond the condition of the hepatic cells, and is, therefore, rather unsatisfactory. But when a very thin section of the same liver is made, after having been hardened in a solution of bi-chromate of potassa, or in Mueller's fluid, and sufficiently large for the purpose, a more perfect view, not only of the individual, but also of the relative condition of all the anatomical elements of the whole parenchyma is obtained when examined under the microscope.

In such sections (Figs. 1 and 2) it will be found, as already mentioned, that generally the cells filling up the neighboring capillary meshes of the hepatic venous radicles, present a more or less dark, greenish-brown color, in some cases mixed with a yellowish tint. The same observation is also made, in a number of instances, on the columns of cells bordering on the radicles of the portal vein (inter-lobular.) These colored cells likewise contain fat-globules, though not as large and as numerous as the uncolored rest, showing that the presence of bile-pigment interfered not with their power of absorbing the fat. The nutmeg appearance of the liver, which was observed in a limited number of cases, as mentioned before—and in which on the contrary the inter-lobular veins were found empty of blood—was probably owing to the coloration of these cells surrounding the vessels.

It is asserted by most pathologists that in the process of fatty infiltration there is no fat deposited outside of the hepatic cells, and that the free fat-globules observed are derived from those cells, cut or torn by the knife in the making of the section. This may be true in cases of phthisis and other chronic exhausting diseases, in which the process of fatty infiltration of the liver is slowly progressing, and sufficient time is afforded to the cells to absorb the fat deposited from the blood into the parenchyma of the organ. In yellow fever, however, where this process frequently arrives at its highest degree in a comparatively short time, and where at the same time—as is true in many cases—the protoplasm of the cells itself degenerates, rendering these minute organs incapable of absorbing the whole of the fat deposited from the blood, it is not at all improbable that a portion of this substance becomes lodged in the interstices of the parenchyma. On the contrary, I may now safely assert that this is a fact, for my careful examination of a large number of well-made and prepared thin sections have not only convinced me of the presence of free fat in the interstices of the parenchyma, but also in those of the connective tissue of the capsule surrounding the organ, and that of the portal vessels (Glisson), or between the latter and the neighboring cells of the hepatic lobules.

In some of the yellow fever livers, the outlines of the hepatic cells appear in the microscopical sections less distinct than in sections made from livers affected with fatty infiltration accompanying other diseases. In the latter, when stained and mounted in Canada balsam, the cells and their nuclei, having absorbed sufficient coloring material, appear very distinct in contrast to the large fat globules which they contain. The hepatic cells of yellow fever livers, on the contrary, do not perfectly absorb the carmine or other coloring materials, in consequence of which their outlines and nuclei appear—especially when mounted in Canada balsam—rather indistinct, and the fat globules within do not show in strong relief. Besides this, in some places of the section, the cells, being filled with numerous minute fat globules, have entirely lost their absorptive power, and, therefore, remain colorless. A great number of the nuclei also undergo fatty degeneration.

The condition of the blood vessels and bile ducts of the liver can be most advantageously studied in these thin sections. Contrary to what might be expected from the observations of some of the clinical phenomena of yellow fever, or from the occasional meeting by way of autopsy with a liver presenting the so-called nutmeg appearance, the blood vessels are, in many cases, found empty of blood. The capillaries, of which, in very thin sections, a number of meshes are frequently met with empty of cells, present here and there a finely wrinkled appearance, which is, however, owing to minute longitudinal rugae, artificially produced by the action of the hardening fluid, as well as to their complete emptiness; a fine double contour indicates the wall of the vessel, beset by the numerous nuclei, also of normal appearance. I have not succeeded positively in detecting fatty degeneration in the latter, though, in some instances, they presented a fatty luster. The intralobular veins (radicles of the hepatic) were always found empty, and their walls in a normal condition. As regards the coats of the interlobular veins (radicles of the portal), and the interlobular hepatic ducts, they were found in the same condition; only in a number of cases the lumen of the interlobular veins, and of the finer branches of the artery, were filled with blood corpuscles (Fig. 2).

With all the attention which I particularly directed to this subject, *I failed to discover any product of inflammation, either in the form of a multiplication of nuclei, or of a cellular exudate, or, even, of pus;* accordingly, *the theory of an inflammatory condition of the liver in yellow fever, which has formerly been held, and still is, by a number of physicians, proves to be fallacious.*

As regards the multiplication of nuclei in the connective tissue of the capsule of the portal canals, mistakes may easily be made by the unexperienced observer in the examination of preparations which have previously been hardened in a solution of chromic acid or its salts, as this agent alters the colored blood corpuscles in the radicles of the interlobular veins and their capillaries in such a manner as to resemble small slightly refractive nuclei.

To the condition of the epithelium of the hepatic ducts, also, I directed my special attention, expecting to find perhaps a fatty degeneration of the cells, but though in some of the smaller ducts these elements seemed to present a fatty luster, I would not venture to positively assert it as a fact, as in the other instances they appeared perfectly distinct and normal, and, if cut horizontally, the ducts mostly showed an open lumen. In thin horizontal sections of the larger portal canals, the condition of the larger vessels and ducts, and also of that system of minute simple and racemose (hepatic) glands, connected with and opening into the latter, can be studied with advantage. In examining a number of these sections, I failed to detect anything abnormal, either in the vessels, ducts, or glands; the pathological changes were entirely confined to the anatomical elements of the parenchyma.

Although the process of fatty infiltration extends in most cases throughout the whole hepatic lobule, there are nevertheless certain portions of the latter more affected than others. Thus, it generally appears that the hepatic cells in the immediate vicinity of the intralobular vein are not affected in as high a degree as those of that region of the lobule, situated between the central and peripheral portions, or even the cells bordering on the interlobular veins (Fig. 1.) In thin colored sections, the degree of the infiltration is easily determined by the degree in which the different parts of the lobule are colored. The best colored portion is usually the central, though there are many exceptions to this rule. As mentioned before, there are certain places met with in very thin sections, in which quite a number of capillary meshes are observed almost entirely deprived of their cells. As in the vicinity of such places the hepatic cells are usually very faintly colored, or not all, it appears as if the falling out of the cells from their meshes depended on a degeneration of their protoplasm; besides, uncolored cells ordinarily contain no large fat globules, but are filled with small ones. In such places of the lobule, where the fatty infiltration has taken place in a high degree, a number of the cells present the so-called seal-ring form (Fig. 1), distended by only one large fat globule, while others may contain two or three.

When examining the first yellow fever livers, at the beginning of the epidemic of 1878, my attention was attracted by a singular appearance of the hepatic cells, resembling a division of the protoplasm of these organs into a number of parts, indicated by shady lines. I have since observed it in many instances, and it really appears as if the granular protoplasm of the whole cell were composed of groups of granules, a supposition rendered very probable by the same observation on hepatic cells undergoing fatty degeneration, in which the same grouping of the minute fat globules is observed. However, this is not the only instance in which I observed this phenomenon, for, a number of years ago, I mentioned the fact that the granular substance in the gray matter of the cerebro-spinal axis consists of an aggregation of minute groups of granules. Even upon the protoplasm of a number of colorless blood corpuscles of man, I have frequently observed this arrangement of the granules.

The pathological changes in the parenchyma of the liver above described are such as I have observed them in the great majority of those cases of yellow fever which I microscopically examined, and from all I know, they may be regarded as typical. In a limited number of cases, however, I observed some additional changes, which either were owing to a severer form of the disease, or to previously existing affections. Thus, I met with two cases in which the coloring of the columns of cells surrounding the intralobular,—or, in some instances, also those surrounding the interlobular veins,—was of a much darker, almost blackish brown, color than in the above cases. Besides, the colored portions or patches were not only confined to the vicinity of the vessels, but moreover met with in other portions of the lobule; in one case, some of these patches presented even a reddish brown. In all other respects, the parenchyma had undergone the same typical changes as above described.

As in all these cases the color was of a greenish or blackish brown, I presume that it depended upon the presence of bile-pigment, though in those few instances where the patch presented the reddish tint, the latter may have depended on the presence of free hæmoglobin, such as was observed in the following case. Here, namely, the patches were of such a size as to be very easily distinguished by the naked eye, and were confined to the intra-lobular veins, embracing them in their entire length (Fig. 3); they were also sharply defined from the rest of the parenchyma of the lobule. Their color was of a dark golden brown, resembling that of old apoplectic foyers in the cortex cerebri. The capillaries of the discolored portions of the hepatic lobules appeared to be compressed, only small portions of them could be recognized with difficulty. The columns of the cells appeared to have been rendered mis-shapen. In each patch, according to the direction of the section of the lobule, a transverse or longitudinal section of an intra-lobular vein could regularly be seen. Though no blood corpuscles could be discovered in the capillaries, there remains no doubt that the coloration of these portions of the hepatic parenchyma was caused by an extravasation of free hæmoglobin, derived from the colored blood corpuscles, through the walls of the vessels, and finally absorbed by the hepatic cells. Small extravasations of free hæmoglobin are observed here and there in sections of other yellow fever livers, and, as I shall show hereafter, also in other organs; but it is not probable that they often occur to such an extent as in the case last described. Nevertheless, at the commencement of September, 1879, I performed an autopsy upon a fatal case of yellow fever at the Charity Hospital, in which the pathological changes in the parenchyma of the liver were of a much graver character than I had met with before in this disease. The patient, a male, died at 9 o'clock A. M., having vomited a quantity of genuine black vomit three hours before his death; the autopsy was commenced two and a-half hours afterwards. The clinical history of this case, together with the characteristic pathological changes in the kidneys and other organs, as revealed by the microscopical examination, left no doubt about the true character of the disease.

The liver in this case was found rather below the ordinary size, and presented a peculiar aspect. The change of color of the surface—depending on the presence or absence of blood in he intra-lobular and inter-lobular blood-vessels, or on the presence of fat in the hepatic cells—instead of presenting to the eye a uniformity of character over the whole organ, differed considerably in different parts. A considerable portion of the upper surface of the right lobe had a bluish color, similar to that of an ordinarily congested liver, such as is frequently observed in malarial disease, while in other portions the congestion seemed to be confined to the finest inter-lobular radicles of the portal vein. In these portions, also, the parenchyma showed the pale yellowish tint, though not so distinctly as is usually seen in this disease. By a closer inspection, however, numerous portions in the form of larger and smaller well-defined patches, ranging in size from that of a hepatic lobule to a ten-cent silver coin, or even larger, of a decided yellow color and without traces of congestion, were discovered, especially upon the upper surface. And, furthermore, in the bluish congested portion of the right lobe, dark, bluish or reddish-brown patches, irregular in size and form, and having the appearance of extravasation of blood, were also noticed. A section through one of the patches showed that they extended in an irregular form into the substance of the organ to the extent of about one inch or more. The consistency of this liver was rather below the normal. A microscopical examination of the fresh parenchyma from the vicinity of the dark patches showed the simultaneous existence of atrophy and fatty degeneration of the protoplasm of the hepatic cells, together with a slight fatty infiltration, consisting of large fat globules.

After portions of the organ had been sufficiently hardened in Mueller's fluid, thin sections were made of different portions of the organ, and colored with carmine, the examination of which disclosed the extraordinary changes, to be now described, which had taken place in the parenchyma of this liver.

To get a correct idea of the whole pathological process which had been going on in this liver, would require a minute description of every detail, with a discussion too extensive for our present purpose. Therefore, a brief sketch of the condition in which the parenchyma was found must suffice. At the first glance through the microscope, upon a section through one of the dark bluish patches and made vertically to the surface of the organ, the anatomical elements of all the hepatic lobules contained in the section appeared to be in a chaos and confusion; for in some places round patches, colored by carmine, were noticed, while in others certain curved columns of cells of a lighter or darker brown, or others uncolored, were also observed. In many places between these portions the cells had disappeared, and through the empty spaces they had left, the field of the microscope could be discovered. The whole parenchyma bordering the inner surface of the general capsule of the upper surface of the liver, contained in the preparation, was, to a considerable depth, found to have assumed a brown color, deepening in shade almost to black, in proportion to the thickness of the section. By repeated examinations with lower and higher powers of a considerable number of sections, I was enabled to study the condition of their individual elements and parts throughout, and, thus far, arrived at the following conclusion. Accordingly, the pathological changes observed in this case, must have been preceded by a hyperæmic condition of the utmost intensity, not only of all the branches of the portal vein, but also, as the examination shows distinctly, of the capillaries of the peripheral portions of the lobules. The intensity of this hyperæmia was such as to induce the hæmoglobin to leave the blood corpuscles, and pass through the walls of the vessels into and between the neighboring cells, a phenomenon which is usually observed in association with a stasis of blood in the vessels. But, besides this extravasation of the coloring material of the blood, a rupture of some of the capillaries must also have occurred, as, in many places, great numbers of blood corpuscles were found located between the atrophied cells. During this state of congestion of the portal veins, the circulation of the blood through the remaining portion of the parenchyma appears to have been mainly carried on by the branches of the hepatic artery, particularly by those arising in the substance of the liver, and passing to the general capsule—rami capsulares arteriosi—which, after extensively anastomosing with each other, give rise to other branches returning into the substance of the organ, and terminate in the capillaries of that portion of the parenchyma situated beneath the capsule. In the sections, these vessels are well displayed, and, as it appears, enlarged in their caliber. As a consequence of the pressure produced by the congested portal veins and their capillaries, and, furthermore, of the simultaneous diminution of the nutrition of the hepatic cells, atrophy of the protoplasm of the latter occurred, not only of those of the peripheral portion of the lobule, but also of those of the center, surrounding the terminal branches of the hepatic veins. Thus, in examining the central portion left in a number of lobules, it appeared as if these cells consisted entirely of nuclei, each surrounded by only a very thin layer of protoplasm. Notwithstanding this atrophied condition of the hepatic cells, however, the whole mass appeared perfectly colored by the carmine, showing that fatty degeneration had, as yet, not taken place in this portion of the lobule. In most of the lobular centers a transverse, or also a more or less longitudinal section of an intra-lobular vein was seen, the lumen of which, in most cases, was filled with a fibrinous thrombus; the walls of these vessels were thickened and contained numerous small spindled-formed cells. In directing my attention to the peripheral portions of the lobules, I could distinctly recognize the form of the capillary meshes, which presented a lighter or darker brown color, the same as is generally observed on blood vessels distended with blood after having remained for some time in a solution of chromate of potassa and subsequently colored with carmine. But the diameter of the capillaries forming these meshes appeared much increased, owing to being still surrounded by a layer of hepatic cells, though the center of the mesh was more or less empty. With an adequate amplification, the blood corpuscles in the interior of these vessels could be well discerned, together with those which had escaped into the surrounding cells, colored brown by the hæmoglobin escaped from these vessels. But there were also here and there empty meshes met with, of which the atrophied cells surrounding the capillaries were colored only by the carmine, showing that they had not absorbed hæmoglobin, nor undergone fatty degeneration. In other places, similar curved columns of cells were observed, which, lying parallel with the periphery of the lobule, and, separated by empty spaces, appeared in the form of successive strata. It would be difficult to describe the details of the destructive process by which these appearances of the parenchyma were produced, though it is obvious that the empty spaces were formed by the degeneration and breaking down of the hepatic cells of this portion of the lobule. Many of these columns, also, were uncolored, their cells having undergone fatty degeneration. Finally, in some places, every trace of an arrangement into lobules appeared to be obliterated, the remaining elements representing a confused mass of degenerated cells, broken here and there by an empty space, or by an isolated section of an intra-lobular vein. Sections of portal vessels were also observed, but their structure appeared indistinct and thickened. The greater number of these represented interlobular branches of the hepatic artery, highly colored with carmine. Sometimes, long columns of carmine-colored, though atrophied, cells were encountered, which, as I suppose, consisted of an arterial branch, surrounded by a layer of cells having, perhaps, received their nourishment from this vessel, preventing their degeneration.

From the above it will be seen that the relative pathological condition in which the anatomical elements of the lobules of the atrophied portions of this liver were found, does not exactly correspond with the condition of the same elements observed in the so-called yellow atrophy of this organ. In the fatty degeneration met with in both instances, as well as in some other minor points, however, a certain resemblance evidently exists. The view, entertained by *Rindfleisch*, that yellow atrophy depended upon an infectious poison, may be true in the case above described.

As regards the other less congested portions of this liver, which formed the greater part of the organ, they were found in the same condition as has been described as "typical" of yellow fever. However, the fatty metamorphosis, observed in this case, was a true fatty degeneration of the hepatic cells; all other elements, as capillary and other vessels, etc., were in a perfectly normal condition; in some form of these capillaries blood corpuscles were observed. There was no atrophy of the cells, they had retained their normal size, but were filled with minute fat globules, which exhibited very distinctly the arrangement in minute groups of which I have spoken above. Only in a comparatively small number of these cells, two or three larger fat globules—though not very large—were observed.

In connection with the pathological changes met in the parenchyma of this liver, I cannot forbear to mention a singular discovery which I made in sections made from those dark bluish portions, and relating to the presence of the mycelium of a very minute parasitic fungus, which extended its numerous filaments throughout the parenchyma of this particular portion of the liver. Not being, myself, an enthusiastic supporter of the so-called germ theory, I would have passed this observation without mention, if the circumstances connected with the presence of this fungus in such an unusual place, would clearly show that the minute plant found its way into the liver accidentally after death. But this is not the case, as the chances for such an accident were quite limited. The autopsy was made two and a half hours after death. The liver, being one of the first organs removed from the body, was put into a basin, and covered with large pieces of ice. There it remained about two hours, when certain parts of it were cut out, and, after being cut again into small cubes of about one to one and a quarter inch diameter, were put, together with pieces of kidney and other organs, into a sufficient quantity of Mueller's liquid. On the second day after, the fluid was removed, and the same precaution was twice more taken before the pieces were hardened enough to be transferred into a fifty per cent. mixture of alcohol and water. A few days after the pieces had been put in Mueller's liquid, I examined some small sections made by hand from one of the pieces belonging to that dark bluish portion of the liver above described, and observed, especially at the thin edges of the section, the fungus consisting of a mature spore with two germinating filaments arising from it at opposite poles. Each filament was bordered by a fine double contour and its interior filled up by a distinct row of granules. At the first sight of these elements, I must confess that I knew not what to make of them, until I met with a bifurcating filament, and observing, furthermore, a peculiar luster on some of the granules in the interior, I began to suspect them to be of a fungous nature. Though these elements might be confounded with long spindle-form cells, a closer examination would easily show a difference existing in the details of both. When the pieces were sufficiently hardened, I was enabled to study the further details of this fungus on the thin sections made by the aid of the microtome. From these examinations, I found that it belonged to one of those families the most simple in construction. The elements which I first observed along the edges of the section, or floating about, represented germinating spores, each of which had given origin to two or three filaments of about four, five, or six times the length of the former. A sporangium was formed upon the terminal points of these filaments, containing about two or three sporules, the discharge of which I witnessed in a number of instances, as also the detachment of the sporangium from the filament. In many specimens, the filaments presented a number of varicosities in their course, sometimes only on one side, almost resembling suckers; in some of them the base was much thicker than the point, and filled with granular protoplasm which had entered from the mother spore; I have also seen the disintegration of the latter. In other instances, again, I observed secondary filaments arising from the first; and likewise bearing a sporangium, or even communicating with a filament of a mother spore. Not being sufficiently versed in systematic mycology, I have failed to classify this fungus, though I have consulted some standard works on the subject. Judging from its mode of fructification, however, it seems to resemble more the Peronospores than any other family.

The presence of this fungus in the parenchyma of this liver, of course, cannot be regarded as an unusual phenomenon, for it might be supposed that it was primarily contained in the hardening fluid, whence it made its way into the pieces of liver. But, then, it would most likely also have invaded all other pieces, not only of the same liver, but also of the other organs contained in the same jar. This, however, was not the case, for, with the exception of the dark bluish atrophied portions infected by the fungus, no trace of the latter could be discovered in those pieces of the same liver affected only by fatty degeneration, or in the pieces belonging to other organs. The only explanation, therefore, left to account for the presence of the fungus in the place mentioned, is, that it was primarily contained in the Mueller's fluid—though this was freshly prepared—and that it selected particularly these disorganized atrophied portions of liver as the best nidus for its development. Whether it was possible that the fungus, or its spores, penetrated by some way or other into the portal circulation, and finally became lodged in some of the interlobular veins, giving rise to the intense congestion and ensuing atrophy of the parts above described, I must leave to the germ theorists to decide. At any rate, I regard the observation sufficiently interesting to be worthy of report, although I entertain not the slightest idea of its standing in any relation with the pathology of yellow fever.

The pathological changes in the parenchyma of the liver, characteristic of yellow fever, as described in the preceding pages, are equally observed in cases where this disease supervened upon another, accompanied by organic changes in the tissues of the same organ, a coincidence which may specially occur in cases of cirrhosis of the liver. An illustrative case of this kind, sufficiently interesting to be cited, I had an opportunity to observe and to examine after death during the epidemic of 1878. A young man of small stature and delicate build, an Italian, was admitted to one of the wards of the Charity Hospital. As I was present at the time, I had occasion to examine him myself. Finding him in an unusual state of nervous exhaustion—for he could hardly walk—with an intensely jaundiced skin, and a very feeble pulse, but otherwise—as could also be gathered from his history—no symptoms of yellow fever about him, I regarded the case as one of chronic malarial fever. As he could speak neither English nor French, himself, it was ascertained by an interpreter that he had been sick for two months, and had been staying with the "Spanish Colony,"—as a portion of the Spanish and Italian population of New Orleans, who, during the epidemic, had left the city to encamp several miles above near the bank of the Mississippi, was called,—whence he came to the hospital. Though at one time slightly improving from his nervous exhaustion, this patient eventually contracted the yellow fever in the hospital, from which he died. The autopsy revealed the following condition of the organs. Not only the skin, but all other tissues in the interior of the body were intensely yellow throughout. The lungs, with the exception of some old slight adhesions to the walls of the thorax, presented a healthy appearance. The heart, also, appeared normal, though the fluid contained in the pericardium was yellow. The liver was enlarged, hard, and granular, showing its cirrhosed condition by gritting under the knife when cut; its capsule was thickened, and its vessels and bile ducts were congested, bile issuing from the cut ends of the latter. The walls of the gall bladder were œdematous. As the patient had thrown up black vomit not long before he died, the stomach was found empty, though its mucous membrane presented that condition of congestion peculiar to yellow fever. The spleen was greatly enlarged, five or six times the bulk of a normal spleen, and also gritted under the knife when cut. The pulp was of a dark color, while the trabeculæ appeared white and enlarged; the capsule of the organ was thickened. The peritoneum covering the diaphragm, liver, stomach, spleen, pancreas and transverse colon, was much thickened, having glued these organs to each other, thus showing that a chronic peritonitis had been existing. The kidneys were greatly enlarged and much congested. The veins of the intestines were filled with blood, though otherwise these organs had a normal appearance. In the cranial cavity the dura mater was yellow, but otherwise healthy, though the vessels of the pia mater were much congested; the arachnoid membrane was found opaque in some places. The cerebral and basilar arteries with their branches were filled with blood. The substance of the brain appeared œdematous, and the ventricles were filled with a yellow fluid. The corpora striata appeared rather pale, while the thalami optici were very white; the superficial vessels of these bodies were filled with blood. In a section of the cerebral hemispheres, the medullary substance appeared very white, the cortical layer pale gray.

The microscopical examination of thin sections of the liver showed that the cirrhosis in this case existed in a considerably high degree. The capsule of Glisson, undergoing the usual hyperplastic metamorphosis, represented now a connective tissue of a coarser and stiffer appearance, than when in its normal condition, though no proliferation of nuclei or spindle cells could any more be observed. The cicatricial tissue formed had encroached upon the lobules, and, in many places, extended into their interior, separating portions of them, which, in the section, appeared in the form of small islands. The hepatic cells at the periphery of the lobules presented a greenish brown color, while minute masses of black pigment were here and there observed deposited between the hepatic cells. The central portions of the lobules, or, also, the portions intermediate between these and the peripheral, were invariably found affected with fatty infiltration (Fig. 4.) This condition existed in a considerably high degree, for most of the cells of the affected portions presented the seal-ring form. In the newly formed tissue of the capsule of the portal vessels the remains of blood vessels were also observed.

Very frequently, especially in cases of drunkards, cirrhosis of the liver is found associated with fatty infiltration. In these cases, however, as in those of ordinary fatty infiltration without cirrhosis, the infiltratory process almost always commences in the hepatic cells at the periphery of the lobule, while in yellow fever, as we have seen, the process appears to commence, or to attain at least its highest degree, near the center, or in portions of the lobule situated intermediate between the latter and the periphery.

[TO BE CONTINUED.]

